# Supplementary material for: Atomistic Study for the Tantalum and Tantalum–Tungsten Alloy Threshold Displacement Energy under Local Strain
Source: Int J Mol Sci. 2023 Feb 7;24(4):3289. doi: 10.3390/ijms24043289 (PMC9965197; doi:10.3390/ijms24043289)
Supplement: Supplementary file 1 [file ijms-24-03289-s001.zip › ijms-2179595-supplementary.pdf]

# Supplementary materials

# Atomistic Study for the Tantalum and Tantalum-Tungsten Alloy Threshold Displacement Energy under Local Strain

by Mohammad Bany Salman [1], Minkyu Park[1], and Mosab Jaser Banisalman [2], *

[1] Virtual lab Inc., 38 Wangsimni-ro, Seongdong-gu, Seoul 08826, Korea

* Author to whom correspondence should be addressed.

**Equations that used to calculate the formation energies in Table S1 as follow:**

In pure Ta the defect formation energy can be evaluate as per the following equation:

$$E_{df}^{f}=E_{df}\ \text{-}E_{per}\ \frac{N_{df}}{N_{per}} \qquad \text{(S1)}$$

Where $E_{df}$ is the total energy of supercell that has defect (monovacancy or SIA), $E_{per}$ is the total energy of the pure Ta supercell, $N_{per}$ is the total number of atoms in perfect supercell. $N_{df}$ is the number of atoms in supercell has defect (it will be ($N_{perfect}$+1when defect is SIA), and ($N_{perfect}$-1 when defect is monovacancy).

The formation energy of the Ta-W mixed defects ($\mathrm{E_{Ta-W}^{f}}$) expresses as follow:

$$E_{Ta-W}^{f}=E_{int}^{Ta-W}-E_{(Ta,w)}\ \text{-}E_{perfect}\ \frac{1}{N_{perfect}} \qquad \text{(S2)}$$

Where $E_{int}^{Ta-W}$ represent total energy of system has (N-1) Ta atoms, and one Ta-W dumbbell. $E_{(Ta,w)}$ is total energy of Ta-W supercell, in which one atom of the Ta structure deducted and one W atom are contained.

$E_{sia}^{Ta-W}$ is the total energy of system having Ta-W dumbbell. Ex is the total energy of Ta-W supercell, in which one W atom added to the pure Ta supercell and one Ta deleted. **Eq.2** follows the definition of the composed alloy defected stated in ref [43].

The binding energy of Vacancy-W ($E_{v-W}^{b}$) is formulated as following the description in ref [44]:

$$E_{v-W}^{b}=E_{(Ta,w)}+E_{v}-E_{w-v}\ \text{-}E_{per} \qquad \text{(S3)}$$

$E_{w-v}$ Represent the total energy of Ta-W supercell including N-2 Ta atoms, one W atom, and one vacancy.

The binding energy between Ta-SIA and W atom calculated using:

$$E_{v-W}^{b}=E_{(SIA\ of\ Ta)}+E_{(Ta,w)}-E_{Ta-SIA+W}\ \text{-}E_{perfect} \qquad \text{(S4)}$$

$E_{(SIA\ of\ Ta)}$ is the total energy of the system composed of one Ta SIA (system with i.e., N+1 atoms of Ta)

where $E_{Ta-SIA+W}$ is the total energy of system containing (N-1) Ta, one W atom as well as most the most stable Ta-Ta interstitial i.e., <111> dumbbell pair.

**Table S1**. Summary of the key properties of W as the point defects in bcc bulk Ta using the present potential, in comparison with the calculations of DFT method. The following values are listed: the substitutional formation energy of W atom in structure contains 128 Ta atoms. The binding energy of two substitutional W atoms ( $E^{b}_{1,2}(W-W)$, the binding energy between W atom and vacancy ( $E^{b}_{1,2}(W-vac)$ ), the formation energy of Ta-W mixed dumbbell ( $E^{f}_{<ijk>}$(Ta-W)). The units of the energy are all given in eV.

| **Values when structure is (Ta-W)** | **By DFT (eV)** |
|---|---|
| $E^{f}_{sub}$(Ta-W) | -0.15 |
| $E^{f}_{<111>}$ (Ta-W) | 4.024 |
| $E^{f}_{<110>}$ (Ta-W) | 4.831 |
| $E^{f}_{<100>}$ (Ta-W) | 5.281 |
| $E^{b}_{1nn}(W-W)$ | -0.135 |
| $E^{b}_{2nn}(W-W)$ | -0.048 |
| $E^{b}_{1nn}(W-vac)$ | -0.297 |
| $E^{b}_{2nn}(W-vac)$ | 0.11 |

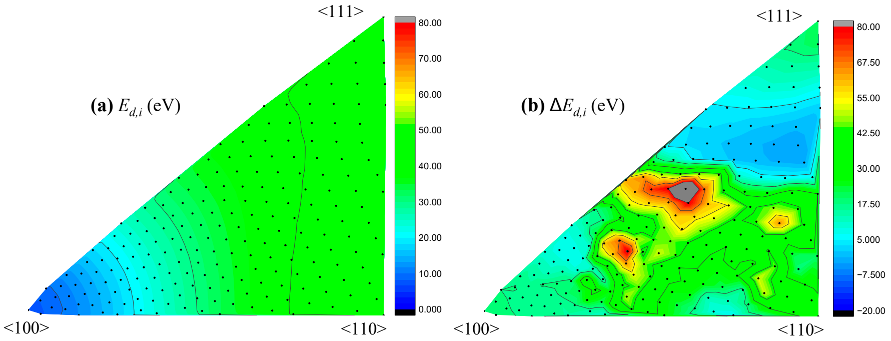


**Figure S1.** Comparison between the theoretical model by Jan and Seeger and the MD calculation results for Ta. (a) Direction dependence of the Ed,i value of the theoretical estimates. (b) Differences between the theoretical estimates and the MD calculations ($\Delta E_{d,i} = E_{d,i@theory} - E_{d,i@MD}$).

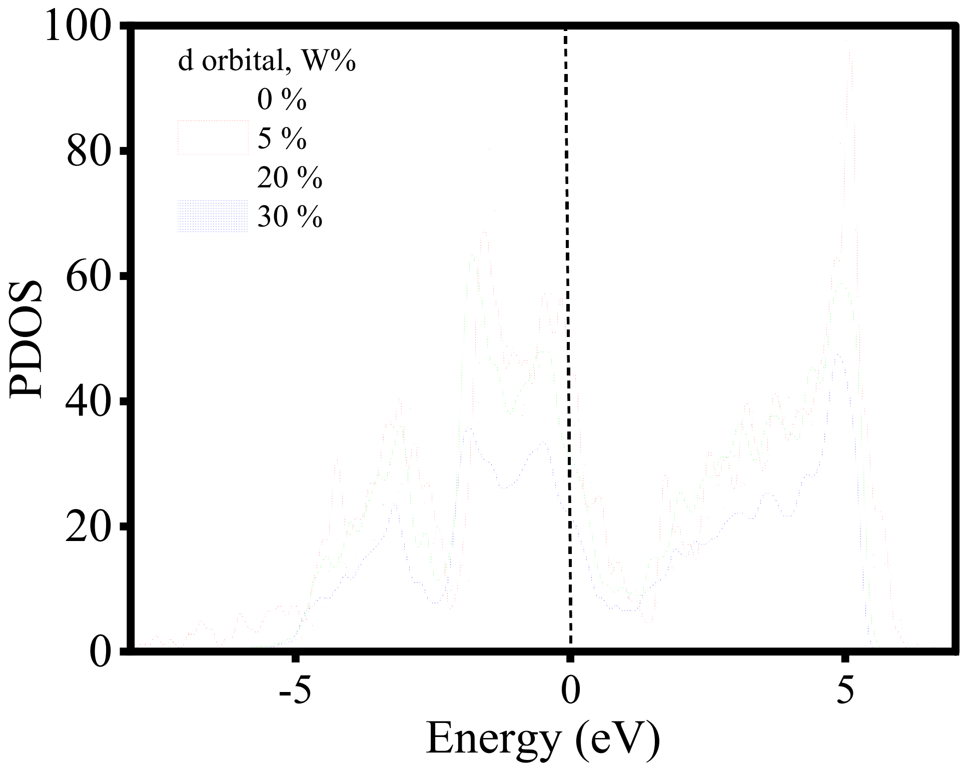


**Figure S2.** The PDOS of the Ta and Ta-*x*W alloy (x=0, 5, 20, and 30 %) alloy. The Fermi level is set to zero. To understand how the electrical structure of Ta alloys changes as a function of W concentration, it is appropriate to investigate how the average number of electrons per atom changes through the DOS and PDOS evaluation. DOS behaves differently when Ta is alloyed with

W because Ta has less valence electrons than W, as illustrated in Figs. S3. The Fermi energy shifts toward the minimum of DOS as the concentration of W increases, and bonding in the alloys becomes more covalent and less metallic. Although the trend of 5% W is relatively like that of pure Ta, the 5% alloyed structure exhibits more metallic properties than the pure Ta structure, which may explain why this ratio has the lowest TDE. The metallic bond shall be weaker as compared to the covalent bond that could explain lower TDE resulted by the 5% ratio.

**Survived number:**

<100> FP=319

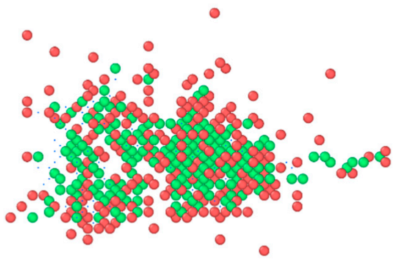

<321> FP=439

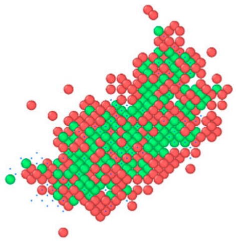

**Survived number:**

<321> FP=16

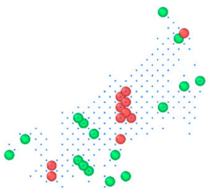

<100> FP=21

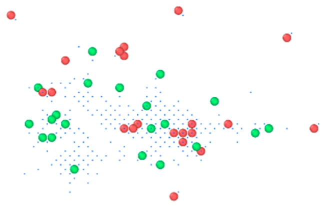

**Figure S3.** a comparative study for PKA evolution when soft or complex direction applied. The PKA shows higher number of collisions with surrounding atoms once move in <321> but most energy will be dissipated and finally less FPs are formed. While when moving with <100> direction smaller chances of interaction with surrounding atoms but higher energy deposited in each collision thus more FPs resulted.

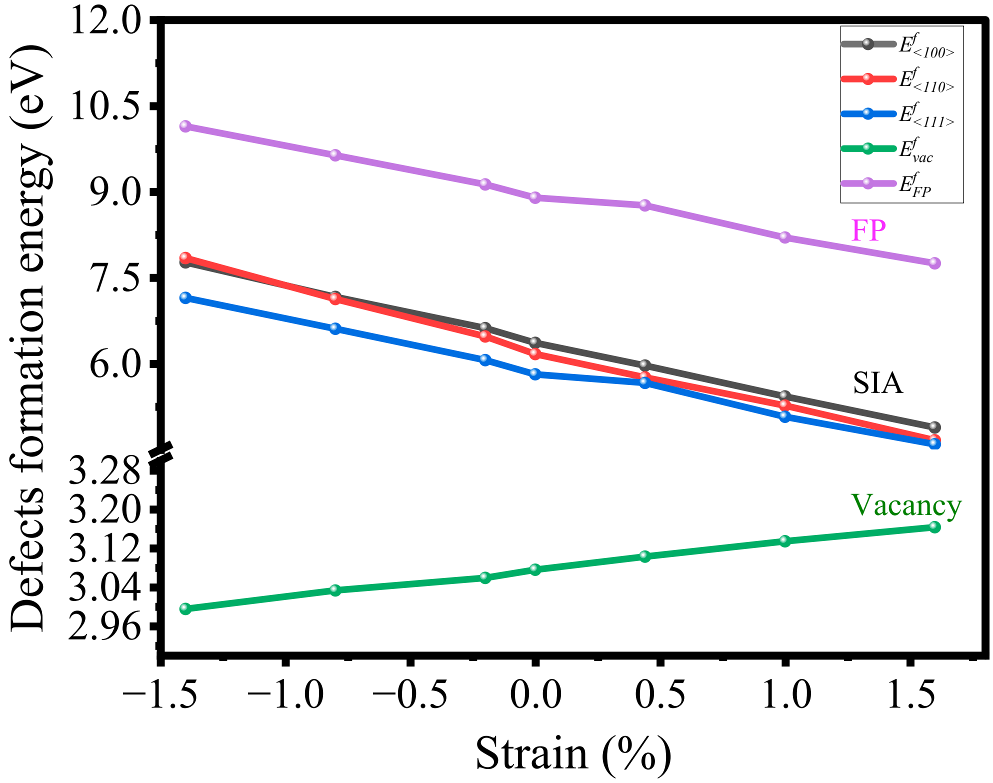


**Figure S4.** The formation energy of defects for Pure Ta once applied to strain from compression to tension.

When the strain amplitude is the same, the effect of strain on defect generation by atomic collision is almost proportional to the volume change (not the strain type), i.e., shear stresses have almost no effect, and hydrostatic deformation has a larger effect than uniaxial deformation, which is why we chose hydrostatic deformation over other strain modes [45].

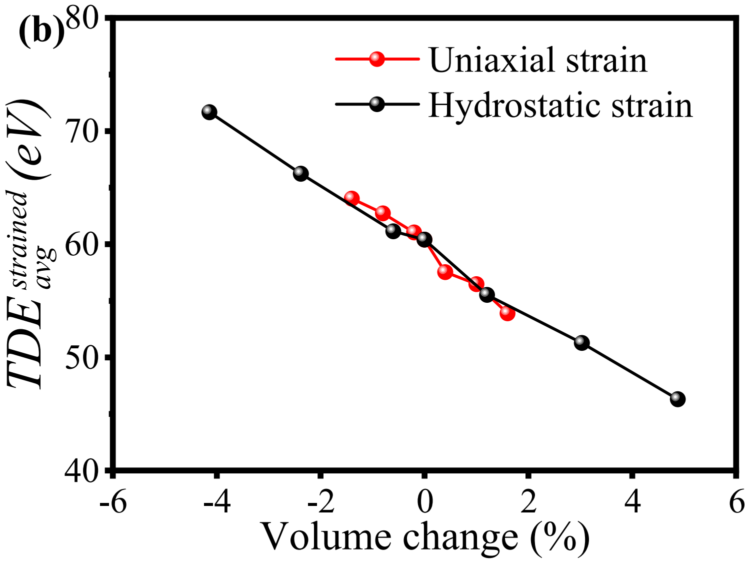


**Figure S5.** $\boldsymbol{TDE_i^{strained}}$, uniaxial and hydrostatic strain in response to the resulted volume change in correlation with TDE for pure Ta.

**References:**


[43] Y.W. You, X.S. Kong, X. Wu, C.S. Liu, Q.F. Fang, J.L. Chen, G.N. Luo, Clustering of transmutation elements tantalum, rhenium and osmium in tungsten in a fusion environment, Nucl. Fusion. 57 (2017). https://doi.org/10.1088/1741-4326/aa70b2.

[44] Z. Cao, M. Pan, K. Hu, Z. Huang, Y. Lv, S. Wen, Y. Zhao, H. Deng, Effect of titanium on the precipitation behaviors of transmutation elements in tungsten-titanium alloys from first-principles calculations, Fusion Eng. Des. 158 (2020) 111673. https://doi.org/10.1016/j.fusengdes.2020.111673.

[45] B. Beeler, M. Asta, P. Hosemann, N. Grønbech-jensen, Effects of applied strain on radiation damage generation in body- centered cubic iron, J. Nucl. Mater. 459 (2015) 159–165. https://doi.org/10.1016/j.jnucmat.2014.12.111.
